# Why is it So Difficult to Choose Safer Alternatives for Hazardous Chemicals?

**DOI:** 10.1289/ehp.120-a280

**Published:** 2012-07-02

**Authors:** Valerie J. Brown

**Affiliations:** **Valerie J. Brown**, based in Oregon, has written for *EHP* since 1996. In 2009 she won a Society of Environmental Journalists’ Outstanding Explanatory Reporting award for her writing on epigenetics.


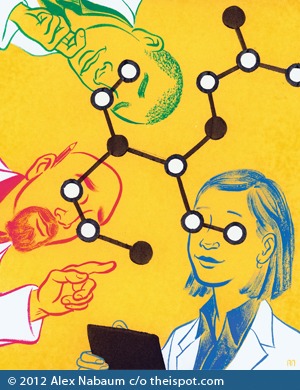
The discovery of persistent, bioaccumulative, and toxic flame-retardant chemicals everywhere from animals north of the Arctic Circle^^1^^ to the breast milk of California women^^2^^ has bee]n a cause for considerable concern. Alternative flame retardants were introduced to replace these chemicals,^^3^^ but investigators had not even produced the first empirical data on the substitutes’ metabolic fate and toxicity before emerging evidence indicated they, like their predecessors, were accumulating rapidly in the environ-ment. As the postmarket research continues, one wonders: Who, exactly, decides on the replacements for toxic chemicals, and on the basis of what criteria? And why does finding truly safer alternatives seem so difficult?

## In Search of Safer Chemicals: The Case of Flame Retardants

Che use of flame retardants in furniture ballooned after the 1975 implementation of California’s Technical Bulletin 117 (TB117),^^4^^ which required foams used in upholstery to withstand a 12-second exposure to a “candle-like” flame. Since then, many furniture manufacturers have chosen to make all their U.S.-sold products compliant with the standard rather than produce separate product streams for California and the rest of the country.

For many years polybrominated diphenyl ethers (PBDEs) were the flame retardants used most often to comply with standards such as TB117 for furniture and other standards for electronics and industrial textiles.^^5^^ But by 2003 it had become evident these chemicals are highly persistent and bioaccumulative in the environment, and studies were starting to report they may cause adverse health effects. *In vitro* and animal studies have been associated various PBDEs with cancer,^^6^^ hormone disruption,^^7^^^^,^^^^8^^ reproductive problems,^^8^^ neurodevelopmental effects,^^7^^^^,^^^^8^^^^,^^^^9^^ and obesity.^^10^^ Associations have also been reported between PBDEs and metabolic syndrome and type 2 diabetes in humans.^^11^^ U.S. body burdens of PBDEs are as much as 10 times higher than those of Europeans.^^12^^ This is likely because the vast majority of the world’s use of penta-BDE, a commercial flame retardant used chiefly in foam for furniture and mattresses, occurred in the Americas.^^13^^

By the end of 2004 pentaBDE and octaBDE (a compound used in plastic housings for electronics) had been banned in the European Union and voluntarily phased out in the United States by sole U.S. manufacturer Great Lakes Chemical Corporation. Certain uses of a third compound, decaBDE, were banned in the European Union in 2008,^^14^^ and all uses will be phased out in the United States by the end of 2013.^^15^^

As pentaBDE was being phased out, Great Lakes Chemical Corporation (which in 2005 merged with chemical manufacturer Crompton Corporation to become Chemtura) introduced a replacement product known as Firemaster 550®.^^16^^ Fire-master 550 contains the flame-retardant chemicals 2-ethylhexyl tetrabromobenzoate (TBB) and bis(2-ethylhexyl) tetrabromophthalate (TBPH).^^17^^ But the process and reasoning by which TBB and TBPH were substituted for earlier compounds remains somewhat opaque.

In a 2005 report on flame-retardant alternatives the U.S. Environmental Protection Agency (EPA) indicated that the unidentified proprietary components of Firemaster 550 have “low” or “moderate” hazard for human health effects and low hazard for persistence and bioaccumulation.^^18^^ However, the supporting documentation behind these determinations reveals a dearth of hard data.^^19^^ For instance, the sections on “proprietary F” and “proprietary H” (each identified only as a “halogenated aryl ester”) contain no data at all regarding chronic or subchronic toxicity, carcinogenicity, neuro-toxicity, immuno-toxicity, genotoxicity, or effects on reproduction or development; the determinations instead were based on expert judgment. The EPA also estimated the degradation products to be high for persistence, as is now being seen.

The metabolism and potential toxicities of TBB and TBPH are only just beginning to be studied,^^20^^^^,^^^^21^^ although their structural similarities to known toxicants lead some to suspect they may not be entirely benign. For example, TBPH, which is also used as a flame-retardant plasticizer in polyvinyl chloride and other applications,^^22^^ has the same carbon skeleton as di(ethylhexyl)phthalate (DEHP), a reproductive toxicant.^^23^^

The lack of empirical health effects data is troubling, given the rate at which the compounds are accumulating in the environment—doubling every one to two years, by one estimate.^^24^^ TBB and TBPH have been found in sewage sludge on both U.S. coasts,^^25^^ Hong Kong marine mammals,^^26^^ the Arctic,^^27^^ the Great Lakes atmosphere,^^28^^ and common household dust.^^29^^ On 1 June 2012 the EPA announced plans to conduct a risk assessment on TBB and TBPH within the next two years.^^30^^

## Federal Assessments of Potential Alternatives

The EPA has attempted to spur partnerships to promote safer alternatives to chemicals of concern through its Design for the Environment (DfE) program. In 2004 the program introduced a process to identify and evaluate known and potential alternatives that may be substituted for problematic chemicals in some applications. To date DfE has reviewed several alternatives to pentaBDE (for use in furniture) and the flame retardant tetrabromo-bisphenol A (for use in printed circuit boards) and is currently reviewing alternatives to decaBDE (for use in plastics and polymers) and the flame retardant hexabromocyclododecane (for use in polystyrene building insulation).

Flame retardants aren’t the only chemicals for which safer alternatives are sought. DfE is also conducting alternatives assessments for bisphenol A in thermal paper and certain phthalates in numerous applications.^^31^^ In December 2011 the agency’s Significant New Alternatives Policy Program, which deals exclusively with protection of stratospheric ozone, approved three hydrocarbons as alternatives to ozone-depleting chlorofluorocarbons.^^32^^

DfE has also tackled alternatives to nonyl-phenol ethoxylates, the most commonly used surfactants in cleaning products, cosmetics, and other applications. These compounds harm aquatic organisms, decreasing both male fertility and survival of juveniles,^^33^^ and are classified by the European Union as possible human reproductive hazards.^^34^^ In May 2012 DfE released a final report identifying eight alternatives to nonyl-phenol ethoxylates.^^35^^

Although new testing is not conducted as part of DfE, the hazard profiles prepared for alternatives assessments often provide more detailed information about chemicals in commerce than is generally available to the public.^^31^^ The assessment assigns alternatives a level of hazard concern for 17 human health and environmental end points. Data gaps can sometimes be addressed with computer models as well as knowledge of chemical analogs and structure–activity relationships (which predict how chemicals will act based on their structure).^^31^^

This information does come with a lower level of confidence, but without it product manufacturers are left to choose alternatives with little or no guidance whatsoever.^^36^^ In fact, new compounds are frequently selected because they are chemically similar to their predecessors and thus will likely preserve their desirable qualities—and, as an unintended consequence, their undesirable ones, says Joel Tickner, a professor of environmental health at the University of Massachusetts Lowell

Because DfE has no authority or budget to require or conduct new testing, its alternatives assessments contain only information that is available in the literature or from manufacturers or that can be modeled. In some cases this information is very limited—a reflection of the status of toxicological testing under the Toxic Substances Control Act (TSCA) of 1976, which grandfathered all chemicals on the market as of 1979 without required testing unless the EPA issued a regulation, Tickner says. TSCA also allows new chemicals to come to market with little or no required testing. According to the EPA, fewer than half the chemicals proposed for commerce have undergone toxicologic testing by their manufacturers.^^37^^

Numerous attempts to update the 36-year-old TSCA legislation have failed. At this point, the law is so ineffectual, says Indiana University distinguished professor of chemistry Ronald Hites, who has tracked the spread of flame retardants in the Great Lakes region,^^28^^ that he has heard it referred to as the “Toxic Substances Conversation Act.” Although a bill to revise TSCA has been introduced by New Jersey senator Frank Lautenberg,^^38^^ few people expect it to pass anytime soon.

## Taking Action Closer to Home

In the absence of political will for federal chemicals policy reform, smaller governmental bodies are acting independently. For example, in January 2011 agencies from nine states formed the Interstate Chemicals Clearinghouse to collect and share information about reduction of toxic chemicals, including a “Safer Alternatives Assessment Wiki” and a searchable state-level chemicals policy database.^^39^^ And California is embarking on what is probably the most comprehensive chemicals regulation in the United States, an effort known as the Safer Consumer Products regulations.

Passed in 2008, the Safer Consumer Products regulations were set to take effect in January 2011 but are still in a round of revisions and public comment.^^40^^ At press time, release of the newest draft regulations was imminent, according to Charlotte Fadipe, a spokes-woman for the California Department of Toxic Substances Control (DTSC). Fadipe says the DTSC hopes to issue the final regulations in the fall of 2012.

Under the regulations the DTSC will develop and prioritize a list of “chemicals of concern” and the “priority products” that employ them based on various human health and ecological criteria as well as likelihood of exposure among potentially vulnerable populations. Any party that manufactures, imports, and/or sells priority products or components of products that contain chemicals of concern will be required to conduct an assessment, evaluating the alternative’s chemical and potential life-cycle hazards, potential exposure scenarios, and economic and performance characteristics. If the DTSC requires adoption of an alternative after the assessment is complete but the chemical remains in the product, the responsible party will be placed on a public Failure to Comply List, and the product will be banned from sale in California within a year.^^41^^

Companies that make and sell consumer products are being pushed by their customers to be more transparent about the chemical content of those products and to use safer alternatives; in turn, they pressure the suppliers of their materials for safer ingredients. Tickner directs the Chemicals Policy and Science Initiative, a program of the Lowell Center for Sustainable Production that offers manufacturers, retailers, regulators, and other interested parties analyses of specific chemicals, policies, and action plans, as well as training to advance evaluation and adoption of safer chemistry. For instance, an “Alternatives Assessment 101”^^42^^ session was conducted in June 2012 in collaboration with the California DTSC.

Tickner says manufacturers and retailers are thirsty for information and ready to reduce their products’ potential health risks. Citing efforts of companies in the footwear and apparel sector such as Hanesbrands and Nike, as well as the American Apparel and Footwear Association (AAFA), Tickner says, “There’s incredible movement in industry. They’re going in directions you wouldn’t have imagined five or six years ago.”

According to its website, Hanesbrands has identified and analyzed all chemicals used in its products and has prohibited or limited the use of formaldehyde, phthalates, antimicrobials, heavy metals, and perfluorinated compounds.^^43^^ The AAFA also provides its members a list of substances whose presence in finished home textile, apparel, and footwear products is banned or restricted by at least one government around the world.^^44^^

## Pulling the Pieces Together

Yet unless a chemical alternative is cost-effective and accomplishes its functional purpose, no company is likely to adopt it even if it is demonstrably greener than a toxic but effective material in use. John Warner, president and chief technology officer at the Massachusetts-based Warner Babcock Institute for Green Chemistry, thinks industry is loath to invest large amounts of money in safer chemicals research because the definition of “safe” is so imprecise and mutable.

“Anyone who is saying we must ban this molecule because it’s toxic should define what the replacement should do unambiguously,” Warner says. Society and government should come up with “a set of criteria so that we can say to industry, ‘If it can pass these tests, it’s safe.’ I think every company would welcome that in a heartbeat—it would change the world overnight.”

Criteria do exist that can be used to define safer chemicals. The European Union has defined chemicals of very low concern as part of its Registration, Evaluation, Authorisation and Restriction of Chemicals (REACH) legislation,^^45^^ and Clean Production Action, a San Francisco–based nonprofit advocacy group, has developed the GreenScreen™ for Safer Chemicals, which can be used to evaluate chemicals and assign levels of concern.^^46^^ The United Nations’ Globally Harmonized System of Classification and Labeling criteria also offer guidance for assessing alternatives and testing recommendations^^47^^ and form the basis of the DfE program’s criteria.^^48^^

“The problem is not a lack of criteria and test guidelines but rather the cost of doing all this testing and the lack of tools and algorithms for integrating data from multiple sources to make informed decisions,” Tickner says. “The current challenges are much more nuanced than simply a lack of guidance: Is there a way to determine which testing is most critical, for instance, and how should emerging end points, such as endocrine disruption, be evaluated?”

The patchwork of industrial collaborations appears to signal broad, relatively stable stakeholder support for safer chemicals, but some experts feel the process is too slow in view of the tens of thousands of chemicals now in use and the paucity of toxicological data on a large percentage of them. “We seem to be under an anesthetic in terms of the judgments we are making,” says Terry Collins, director of the Institute for Green Science at Carnegie Mellon University in Pittsburgh. Collins likens U.S. political inaction on safer chemical alternatives to the people of Easter Island stubbornly cutting down all their trees even as they knew it spelled their doom.^^49^^

Other observers also stress that choosing chemical alternatives from the library of existing chemicals is not “green chemistry,” per se. Green chemistry entails designing chemicals that are “intrinsically safe,” says Tickner, but “that’s a slow process. In the interim, how do we get to ‘safer’?” He views the various attempts to substitute safer chemicals as better than the alternative of doing nothing while true green chemistry develops.

In the absence of nationwide, comprehensive reform, the efforts now under way to identify and use safer chemical alternatives could be viewed as working out the bugs in policies and practices—the results of a diverse range of voluntary approaches may help determine what works and what doesn’t, making it relatively easy to devise a rational and effective national policy if and when there is a clear political intention to do so. Until that intention manifests, however, it remains possible for alternatives that themselves are persistent, bioaccumulative, and toxic to continue entering widespread use.
